# Spatiotemporal evolution of hedging effects in Asia-Pacific countries amid Sino-US competition: Insights from massive event data

**DOI:** 10.1371/journal.pone.0317308

**Published:** 2025-01-15

**Authors:** Qiyue Hu, Lihua Yuan, Bin Liu

**Affiliations:** 1 Center for Geodata and Analysis, Faculty of Geographical Science, Beijing Normal University, Beijing, China; 2 School of Business, Guangxi University, Nanning, China; UCSI University, MALAYSIA

## Abstract

Facing the pressure of Sino-US strategic competition, countries in the Asia-Pacific region often adopt hedging strategies to minimize risk and protect their interests. If implemented, these strategies could impact relationships between countries and lead to political instability. Owing to a lack of theoretical evaluation frameworks and methods, few studies have examined the implementation effects of hedging strategies adopted by Asia-Pacific countries amid Sino-US competition. This study proposes a novel four-quadrant evaluation theoretical framework, and constructs a Geopolitical Relation Index and a Comparative Relation Index based on the Global Database of Events, Language, and Tone massive event data. Since 2000, the effects of hedging strategies in 19 Asia-Pacific countries against China and the US have been dynamically quantified. The research reveals that Asia-Pacific countries’ dynamic performances over 24 years can be categorized into three groups: significantly closer to China, significantly closer to the US, and swinging. Since implementing the Belt and Road Initiative, countries close to China have deepened their ties, while those aligned with the US have strengthened their ties. Asia-Pacific countries have demonstrated similar characteristics from Obama’s presidency to Biden’s presidency. The results contribute to the dynamic assessment and ongoing monitoring of the execution effects of Asia-Pacific countries’ diplomatic strategies towards China and the US, offering valuable insights for timely refinement of their foreign policies.

## 1 Introduction

As a crucial global economy and political engine, the Asia-Pacific region has significantly influenced the world through its prosperity and stability. However, owing to geopolitical realities and strong US intervention, the Asia-Pacific region has been involuntarily caught in the vortex of Sino-US strategic competition, greatly increasing uncertainty in foreign policy formulation and implementation. This uncertainty negatively affects the relationships between countries and major powers, challenging global sustainable development. Strategic competition has become a prominent feature of Sino-US relations since the beginning of the 21st century, profoundly affecting regional and global geopolitical landscapes. Simultaneously, strategic competition between the US and China will continue for several decades [1–4]. From the Obama-era *rebalance* *to* *the* *Asia*-*Pacific* to the Trump administration’s *Indo*-*Pacific* *strategy* and the Quadrilateral Security Dialogue (QUAD) and Indo-Pacific Economic Framework for Prosperity (IPEF), widely seen as Asia’s version of NATO, the Asia-Pacific region always remains a strategic focal point for the US to contain the rise of China.

As Sino-US relations enter a phase of strategic competition, the regional order has become more complex and uncertain. The Asia-Pacific region faces major challenges in the strategic realignment and diplomatic decision-making between the two major powers. Specifically, there are concerns about the credibility of the States’ security commitments and the possibility that China may significantly influence the region through its growing economic position [1, 5]. Faced with the pressures of Sino-US strategic competition, countries in the Asia-Pacific region often adopt hedging strategies to secure the defense advantages of aligning with the US while while also benefiting economically from China. These strategies cover multiple aspects including politics, economy, security, and military.

How does the relationship between Asia-Pacific countries and the two great powers evolve after implementing these hedging strategies? Is this in line with the expectations of these countries when they first formulate their strategies? While insightful, traditional qualitative analyses are often burdened by inherent subjectivity, which impedes their ability to provide precise and timely assessments of hedging effects. Incorporating a dynamic quantification methodology to evaluate the implementation effects of hedging strategies adopted by Asia-Pacific countries can offer valuable insights for policymakers to monitor and adjust diplomatic strategies to mitigate or avoid regional tensions. Such adjustments promote sustainable development in the Asia-Pacific region. Regrettably, limited analyses have been conducted in this area, partly because of a lack of analytical frameworks, methods, and data. To address this issue, this study uses massive event data to evaluate the effects of strategic behavior in Asia-Pacific countries by constructing a four-quadrant theory framework and quantitative methods and then observes the features of the spatiotemporal dynamic evolution of the effects of Asia-Pacific countries’ strategic behavior. This study also verifies the robustness of the theoretical evaluation framework for assessing the effects of third-party hedging strategies between two great powers.

The remainder of this paper is organized as follows: Section 2 critically reviews the strategic behavior options of Asia-Pacific states vis-à-vis China and the US, the latest progress in quantifying international relations. Section 3 presents the theoretical evaluation framework and quantitative methods. Section 4 presents the main results of the quantitative research, and Section 5 presents the conclusions and discussion.

## 2 Asia-pacific hedging strategy under great power competition

Amid great power competitions, third-party countries usually consider balancing and bandwagoning as primary responses. During the Cold War, several third-party nations, including China, implemented similar strategies in response to US-Soviet rivalry, with China being the prime example. In response to the intense rivalry between the US and the Soviet Union, China adopted various strategies at different intervals. From initially following the Soviet Union’s “leaning to one side” foreign policy, to simultaneously opposing the US and Soviet’s *two*-*line* *strategy*, then to the *single* *line* *strategy* of aligning with the US against the Soviets, and finally to the post-Cold War *independent* *and* *non*-*aligned* foreign policy, China made various choices at different periods to deal with the impact of intense US-Soviet’s competition. Obviously, the strategies adopted by a third-party country to cope with great-power competition are dynamic and diverse. Scholars have proposed theoretical frameworks such as *balance*-*of*-*power* theory [6], *balance*-*of*-*threat* theory [7], *bandwagoning*-*for*-*profit* theory [8] to explain the reasons and mechanisms.

As a representative emerging developing country, China has touched the sensitive nerves of the United States with its rapid growth. To contain China’s rise, the White House has successively implemented the pivot to Asia strategy, the Asia-Pacific rebalancing strategy, and the Indo-Pacific strategy, launched the longest-running trade dispute in history, and deliberately suppressed China’s high-tech industry. Since the establishment of diplomatic ties in 1971, the relationship between China and the US has transitioned from cooperation to strategic competition. In response to the uncertain changes associated with rivalry between the two major powers, several Asia-Pacific nations facing limited strength must dynamically adjust their strategies to mitigate or circumvent potential negative consequences. Recently, the strategic decisions made by these Asia-Pacific nations have garnered scholarly interest. However, traditional balancing or bandwagoning explanations have become weak. Furthermore, the strategic options available to a third country with great power competition are not binary choices. Considering this, analysts have adopted various terms to describe different strategic and diplomatic choices made by different countries, including *omni*-*balancing* [9], *soft* *balancing* [10], *complex* *balancing* [11], *engagement* [12], *accommodation* [13], *great* *power* *balancing* [14] and *hedging* [15–19], among others.

Emerging from the research is the concept of hedging. Hedging is most commonly used in metadiscourse analysis. In recent years, researchers have explored metadiscourse from various perspectives, examining its diversity across different contexts and cultures, leading to more refined classifications. Carrió-Pastor views hedging as devices that mitigate the proposition and protect the face of writers [20]. Ruonan and Al-Shaibani [21] found that hedges such as (*more*) *likely*, *may* (*not*), *may* *be*, *could* *be*, *possible*, and *might* are used to sound less assertive in undergraduates’ abstracts in social sciences. By hedging, speakers/writers are able to set caution and self-deprecation, thereby avoiding controversy caused by assertions [22]. Hedging was introduced into the field of international relations(IR) in the 1990s to explain the way countries interact with each other, often in contrast to balancing or bandwagoning [23, 24].

As uncertainties deepen, weaker powers in Southeast Asia, as elsewhere, are compelled to hedge one way or another [25]. Ideally, the hedging strategy provides smaller powers with insurance against the negative impacts of the uncertain actions of the great power. This understanding implies that hedging behavior is not restricted to political, security, or economic dimensions. Instead, a promising hedging strategy must combine policies and approaches from all three policy areas [26]. It is commonplace to hear that Southeast Asia does not want to have to choose between the US and China [11, 27, 28]. They have long pursued hedging policies that try to juggle their ties with China and the US [29]. Hedging now is one of the most influential concepts to emerge from scholarship on the international relations of the Asia-Pacific in the 21st century [30–42]. Analysts have mainly focused on the meanings, behavior types, and mechanisms of hedging strategies utilized by countries in the Asia-Pacific region.

Although differences of opinion still exist, there appears to be some agreement that hedging refers to a mixed-policy approach. Evelyn Goh defines hedging as a set of strategies aimed at avoiding (or planning for contingencies in) a situation in which states cannot decide upon more straightforward alternatives such as balancing, bandwagoning, or neutrality. She further points out that hedging behavior in Southeast Asia comprises three elements: first, indirect or soft balancing; second,complex engagement; third, enmeshment policy [15]. Kuik argues that hedging is a series of flexible combination strategies adopted by countries to avoid risks and obtain returns [18, 31]. For Alexander, hedging means a type of state behavior that emphasizes relative equidistance in relations with other powers and combines both engagement and containment, which helps states to avoid taking one side at the obvious expense of another [43]. Acharya agrees that hedging is an avoidance of taking sides in the rivalry while watching the competition unfold, but argues that co-engagement is a more realistic description of Southeast Asian countries’ behavior. [44]. Regarding the classification of hedging types or behaviors, scholars have proposed diverse perspectives. Evan views that the hedging strategy possesses both cooperative and competitive dimensions [27]. Matsuda holds that hedging refers to maintaining close economic and diplomatic contact with one country while simultaneously establishing or maintaining security ties with another country [45]. Chinese analysts often use *hedging* *your* *bets* or *playing* *both* *sides* to describe this behavior [46]. The term hedging here denotes a country’s reliance on China for economic purposes and the US for security [47]. In research focused on understanding the mechanisms behind state hedging behavior, academics have suggested various viewpoints. These include considerations of risk aversion [34, 48], the pursuit of national interests [37], external influences such as competition among great powers [49], and domestic factors, particularly the perceptions held by elite groups [31].

Nevertheless, there is still no consensus as to how hedging should be defined and applied. David Martin has criticized the dominant understandings of hedging, arguing that the term remains a vague concept [19, 41]. Furthermore, the hedging strategies adopted during different periods vary by country. Regarding geopolitical scope, there are considerable discrepancies in how countries respond to Sino-US competition and collaboration. Regarding temporal considerations, a country’s strategic responses diverge in different periods [25, 26, 50]. In summary, previous published studies are limited to a qualitative approach and the effects of these strategies, particularly in terms of quantitative analyses within the existing literature, remain unclear. With the rapid advancement of information technology, especially in the realm of artificial intelligence, the availability of data for quantitative research in international relations has significantly increased. This development establishes a robust foundation for quantitative studies concerning national strategic choices. This study develops a four-quadrant evaluation model and quantitative methodology to explore innovative approaches that combine theoretical analysis with massive event data. Based on this analytical framework, the effects of the hedging strategies adopted by Asia-Pacific countries are characterized by their relative relation proximity to China or the US. The main purpose is to address the existing gap in quantitative research related to the effect of strategy implementation.

## 3 Four-quadrant evaluation model and its corresponding quantitative methodology

### 3.1 Definition of hedging strategy

A clear definition of the hedging strategy is essential before embarking on a quantitative assessment in this study. However, just as speakers and writers exercise caution, evasion, and ambiguity through hedging words, this vague quality persists when a nation takes on the role of a hedging initiator. According to Goh (2005), Kuik (2008), Alan (2016) and Koga (2018), hedging is when a country seeks to offset risks by pursuing multiple policy options to produce mutually counteracting effects under high uncertainty and high stakes [31]. Within the Asia-Pacific region, hedgers simultaneously perceive substantial benefits of engaging with China economically while fearing China’s potential to become politically and/or militarily overbearing. They want to maximize current opportunities while protecting against future threats [51]. Thus, Hedging strategies for Asia-Pacific encompass strong engagement with China and the facilitation of a continuing US strategic presence in the region to act as a counterweight or balance against rising Chinese power [16]. This definition implies that Asia-Pacific states pursue a contradictory and fluid mix of strategies [51], reflected in political, economic, security, military, and other fields [52]. This study does not want to continue the debate on the concept of hedging in IR field. Rather, it focuses on the specific effects of hedging strategies adopted by Asia-Pacific countries, along with their temporal and spatial evolution. We attempt to construct a four-quadrant evaluation framework to quantitatively evaluate the relative relation closeness between Asia-Pacific countries and China or the US to reveal the effects of their hedging strategies.

### 3.2 Four-quadrant theory evaluation framework

Owing to the limitations of existing hedging theories and analytical frameworks in evaluating the effects of hedging strategies by third-party countries amid Sino-US strategic competition, it is essential to develop a novel theoretical assessment framework. When one country hedges against another, the most intuitive manifestation is the dynamic relationship between the two countries. Since how a country interacts in global affairs is the most direct reflection of its diplomatic strategies with other countries, geopolitical relationships can capture the effects of hedging strategies for Asia-Pacific countries during different periods of Sino-US strategic competition. Considering that both active and passive actors are in a diplomatic event, this could lead to different perceptions of the relationship between the two countries. The framework takes a third-country perspective to quantify its geopolitical relationships with the US and China to ensure comparability.

The theoretical evaluation framework is based on geopolitical relations. The interactions between the two nations include cooperation and conflict, showing the basis of geopolitical connections between the states. However, it is difficult to accurately understand the dynamic evolution of international relationships when these two aspects are combined. To better develop the theoretical evaluation framework, we propose a Geopolitical Relation Index (GRI), including two dimensions, denoted as *GRI*^(+)^ and *GRI*^(−)^, respectively. A higher *GRI*^(+)^ indicates a closer cooperation. Conversely, the higher *GRI*^(−)^ indicates a more serious conflict. In this study, the GRI of Asia-Pacific states with China and the US are *GRI*_*iC*_ and *GRI*_*iU*_, where *i*, *C*, and *U* represent Asia-Pacific states, China and the US, respectively. We then constructed a Comparative Relation Index (CRI) to measure the relative distance of a country’s geopolitical relationship with China and the US. The formula used is as follows:

$$\begin{array}{c} CRI_{i}^{+}=GRI_{iC}^{\left(+\right)}-GRI_{iU}^{\left(+\right)}, \end{array}$$ (1)

$$\begin{array}{c} CRI_{i}^{-}=GRI_{iC}^{\left(-\right)}-GRI_{iU}^{\left(-\right)}, \end{array}$$ (2)

**Fig 1:**
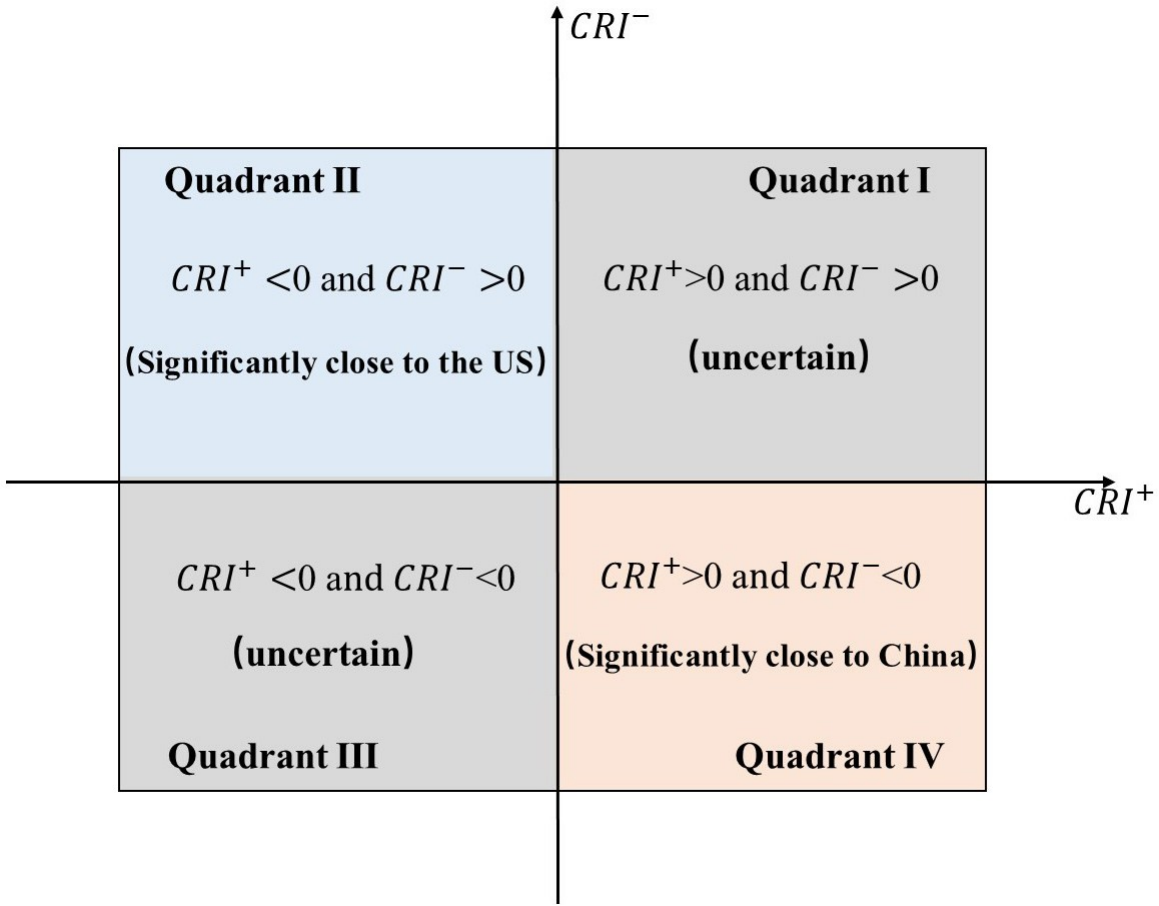
The four-quadrant theory evaluation model based on Geopolitical Relation Index.

Considering whether *CRI*_*i*_ is greater than 0 as the cutoff point, the relative relationship of Asia-Pacific country *i* between China and the United States can be divided into four quadrants, as shown in Fig 1. When $CRI_{i}^{+}$ is greater than 0, it implies the geopolitical relation between country i and China in term of cooperation is better than that of the US; when $CRI_{i}^{-}$ is greater than 0, it implies the geopolitical relation between country i and China in term of conflict is more serious than that of the US. According to this rule, we find that results in the quadrant II and IV are certain, which are significantly closer to the US and significantly closer to China, respectively.

However, the results in the quadrant I and III are uncertainty and require further investigation. Here, we propose another cutoff point. For the quadrant I ($CRI_{i}^{+}>0$ and $CRI_{i}^{-}>0$), if $CRI_{i}^{+}/CRI_{i}^{-}>1$, it indicates that the level of cooperation between country *i* and China or the US is higher than the level of conflict between country *i* and China or the US. We find that country *i* is closer to China than to the US. Conversely, the country *i* is closer to the US. For the quadrant III ($CRI_{i}^{+}<0$ and $CRI_{i}^{-}<0$), if $CRI_{i}^{+}/CRI_{i}^{-}>1$, it implies that the level of conflict between country *i* and China or the US is higher than the level of cooperation between country *i* and China or the US. We find that country *i* is closer to the US than to China. By contrast, country *i* is closer to China.

The four-quadrant theoretical assessment model allows for the evaluation of the relative relationship of Asia-Pacific countries towards China and the US over a certain period. However, determining the exact degree of these interactions can be difficult. To enhance the accuracy of the assessment, we present a new algorithm as detailed below: Case 1: For Quadrant I, the degree closer to China is $CRI_{i}^{+}/\left(CRI_{i}^{-}+CRI_{i}^{+}\right)*100$. Case 2: For Quadrant II, the closeness to China is 0%. Case 3: For Quadrant III, the degree closer to China is $CRI_{i}^{-}/\left(CRI_{i}^{-}+CRI_{i}^{+}\right)*100$. Case 4: In Quadrant IV, the degree of closeness to China is 100%. This enhancement is designed to quantify the relative closeness of the relationship between Asia-Pacific countries and China, ranging from 0% to 100%. A higher value indicates a closer relationship between a given Asia-Pacific country and China in comparison to its relationship with the US. It is important to note that the CRI index serves as a relative metric, and fluctuations in its value do not necessarily indicate a direct strengthening or deterioration of the bilateral relations between country *i* and either China or the US.

### 3.3 Data sources and research methodology

#### 3.3.1 Data sources

International relations are complex and volatile, involving political, economic, security, and military aspects [53]. Conventional analytical techniques, such as case studies of major events, policy analyses, and single-topic studies, are inherently static. This limitation renders them incapable of providing accurate and comprehensive analyses of the dynamic changes in geopolitical relationships. Consequently, it is impossible to evaluate the effects of hedging strategies accurately. The quantification of international relations has become increasingly prevalent based on event data. Analysts engaged in discussions regarding the development and contributions of quantitative research on global international relations and the potential benefits of employing quantitative methodologies in studying foreign affairs [54]. With the rapid growth of big data, quantifying numerous events occurring between countries and statistical methods provides a good way to capture the dynamics of geopolitical relations [55].

The Global Database of Events, Language, and Tone (GDELT) is a massive event database based on the Conflict and Mediation Event Observations (CAMEO) event coding framework [56], and is the most comprehensive event datasets that collects events from most national and local news media worldwide [57]. Each recorded event within the database involves two participating entities, designated as Actor1 and Actor2, where Actor1 typically represents the active participant and Actor2 the passive participant. The dataset encompasses 58 variables, including the Goldstein Scale (GS), number of mentions of an event, the type of event, its geographical location, and the temporal context of the event, among others. The Goldstein Scale represents the degree of cooperation or conflict associated with an event, with scores ranging from -10 to 10,indicating the extremes of conflict and cooperation, respectively. The number of mentions can be regarded as a key indicator to measure the significance of an event. The attributes of the GDELT database render it a valuable resource for quantitative analyses in the field of international relations. Over the past decade, many scholars have utilized the GDELT dataset for research on this subject [53, 55, 57–65], demonstrating the practicality and effectiveness of using massive event data in academic studies of international relations.

According to the definition of hedging in this article, events are the concrete carriers of the implementation of hedging strategies. Asia-Pacific nations adopt hedging strategies concerning China and the US, illustrating a blend of cooperation and conflict across politics, economics,security, and military. Despite facing some criticisms regarding its limitations [66], the GDELT dataset, with its tremendous amount of event records more than any other event datasets, encompasses a diverse array of cooperation and conflicts across multiple domains [67]. This breadth of data offers substantial empirical support for the efficacy of quantitative hedging strategies. Accordingly, we extracted event data pertaining to the interactions between Asia-Pacific nations and both China and the US from the GDELT database, utilizing this information as the foundational dataset for our quantitative four-quadrant analysis framework.

Owing to limited data availability in the GDELT database for certain countries, this study focused on 19 countries in the Asia-Pacific region, as outlined in Table 1. Here, we take these Asia-Pacific countries as the active parties to the event (Actor 1) and China or the US as passive parties (Actor 2). Considering China’s accelerated growth trajectory since the turn of the 21st century, our research encompasses the period from January 1, 2000, to December 31, 2023. The number of events related to conflicts and cooperation of the 19 Asia-Pacific countries with China, and the US was obtained from GDELT, as presented in Table 1. There are substantial disparities in the number of events pertaining to cooperation and conflict among Asia-Pacific countries, China, and the US. To illustrate this difference, we calculated the ratio of the number of cooperative events to that of conflict events. As can be seen, the indicator of Cambodia, Laos, Pakistan, Nepal, and Bangladesh to China is even more than twice that of the US, while the ratio of the Philippines, and Brunei to the US is more than double that of China. However, it should be noted that this variable is insufficient to identify the effects of hedging strategies.

**Table 1:** Summary of the number of events for Asia-Pacific states with China and the US from 2000 to 2023.

Country (Code)	Number of China-related events in Asia-Pacific countries			Number of US-related events in Asia-Pacific countries		
	Cooperation (GS>0)	Conflict (GS<0)	*Ratio* *	Cooperation (GS>0)	Conflict (GS<0)	*Ratio* *
Cambodia (KHM)	33,087	6,268	5.28	19,852	7,980	2.49
Laos (LAO)	15,016	2,103	7.14	6,822	2,117	3.22
Myanmar (MMR)	30,126	8,377	3.6	40,422	15,330	2.64
Mongolia (MNG)	17,634	3,460	5.10	6,641	960	6.92
Bangladesh (BGD)	21,828	3,391	6.44	33,711	13,016	2.59
Pakistan (PAK)	144,589	32,764	4.41	226,528	132,633	1.71
Nepal (NPL)	41,872	8,326	5.03	20,292	5,992	3.39
Malaysia (MYS)	76,871	20,365	3.77	48,035	18,542	2.59
Thailand (THA)	47,462	11,632	4.08	45,630	17,726	2.57
Vietnam (VNM)	80,807	39,907	2.02	144,836	50,034	2.89
Indonesia (IDN)	45,560	14,723	3.09	59,093	20,701	2.85
Philippine (PHL)	107,936	77,714	1.39	140,750	55,428	2.54
Singapore (SGP)	45,263	9,144	4.95	48,185	9,682	4.98
Brunei (BRN)	23,922	11,604	2.06	7,852	1,992	3.94
New Zealand (NZL)	33,254	9,016	3.69	70,187	20,444	3.43
Australia (AUS)	142,468	64,777	2.20	342,646	127,028	2.7
Japan (JPN)	239,025	90,986	2.57	419,509	126,195	3.32
South Korea (KOR)	132,835	32,338	4.11	230,987	66,552	3.47
India (IND)	49,893	21,712	2.30	114,362	43,250	2.64

* Ratio is equal to number of events at the cooperative level over number of incidents at the conflictual level.

#### 3.3.2 Research methodology

Existing international relations studies utilizing the GDELT database incorporate variables such as number of events, average of GS, and the sum of GS. However, the number of events does not consider the varying impacts of different events on bilateral relationships. Furthermore, the GS fails to acknowledge the spatiotemporal differences in the influence of the same type of event. Specifically, the GS indicator has shortcomings in both areas. First, events of the same type are assigned the same GS values in different spaces. For example, when president of the United States visits Vietnam and Germany, similar events are assigned the same GS value. However, the impact of a national leader’s visit to different countries on their relationship should vary. Second, events of the same type had identical GS values at different time points. For example, visits during the reestablishment of diplomatic relations between China and the United States and reciprocal visits during strategic competition between the two countries have the same GS value, which is unreasonable. Thus, it is illogical to apply GS directly as an evaluation measure. The GDELT database provides a variable named the Number of Mentions (NoM), which can be used as a proxy for the importance of events. We introduced this indicator to obtain the GS-modified (GSM), which was calculated using the following formula:

$$\begin{array}{c} GSM=GS*ln(NoM) \end{array}$$ (3)

where the minimum value of NoM is 1, and GSM is the improved indicator. The introduction of the NoM logarithm addresses the limitations of event counts and GS. Additionally, it acts as a filter that automatically removes insignificant events, enhancing the accuracy of quantitative analysis.

We then elaborate on the construction process of GRI. The interactions between two countries consist of both cooperative and conflictual events, prompting us to quantify their relationship from the standpoint of cooperation or conflict. In light of this, we assess the extent to which cooperative or conflictual events impact all occurrences, utilizing the GSM, denoted as $R_{ij}^{\left(+\right)}$ and $R_{ij}^{\left(-\right)}$.

$$\begin{array}{c} R_{ij}^{\left(+\right)}=\frac{\sum_{k=1}^{N^{\left(+\right)}}GSM_{ij}^{\left(k,+\right)}}{\sum_{k=1}^{N^{\left(+\right)}}GSM_{ij}^{\left(k,+\right)}+\left|\sum_{k=1}^{N^{\left(-\right)}}GSM_{ij}^{\left(k,-\right)}\right|} \end{array}$$ (4)

$$\begin{array}{c} R_{ij}^{\left(-\right)}=1-R_{ij}^{\left(+\right)} \end{array}$$ (5)

where k represents event, *i* and *j* represent country, + and − denote as cooperation and conflict, *N*^(+)^ and *N*^(−)^ represent the number of cooperative and conflict events, respectively. However, *R*_*ij*_ does not consider the degree of importance of country *j* in the international interactions of country *i*. Therefore, we should introduce a weight, denoted as *W*_*ij*_.

$$\begin{array}{c} W_{ij}^{\left(+\right)}=\frac{N_{ij}^{\left(+\right)}}{\sum_{k=1,k\neq i}^{N_{c}}N_{ik}^{\left(+\right)}} \end{array}$$ (6)

$$\begin{array}{c} W_{ij}^{\left(-\right)}=\frac{N_{ij}^{\left(-\right)}}{\sum_{k=1,k\neq i}^{N_{c}}N_{ik}^{\left(-\right)}} \end{array}$$ (7)

where, *N*_*c*_ is the number of country. The GRI formula presented in this article is derived from a combination of *R* and *W*. Here, country *i* denotes a nation within the Asia-Pacific region, while country *j* refers to either China or the US.


$$\begin{array}{c} GRI_{ij}^{\left(+\right)}=W_{ij}^{\left(+\right)}*R_{ij}^{\left(+\right)} \end{array}$$ (8)

$$\begin{array}{c} GRI_{ij}^{\left(-\right)}=W_{ij}^{\left(-\right)}*R_{ij}^{\left(-\right)} \end{array}$$ (9)

The impact of various affairs between the two countries on the relationship is long-lasting; thus, selecting an appropriate timescale is particularly important in the quantification study of geopolitical relations. The GDELT database contains four timescales: daily, weekly, monthly, and annually. Noise is present in the daily or weekly data [58]. However, although the duration of an event is not known with certainty, it is rarely longer than one year in general, implying that annual data may not be able to capture short-term interactions [55]. Consistent with previous research, a monthly scale was appropriate for our study.

## 4 Results

### 4.1 Dynamic evolution of Asia-Pacific countries’ CRI with China and the US—A case study of Australia

**Fig 2:**
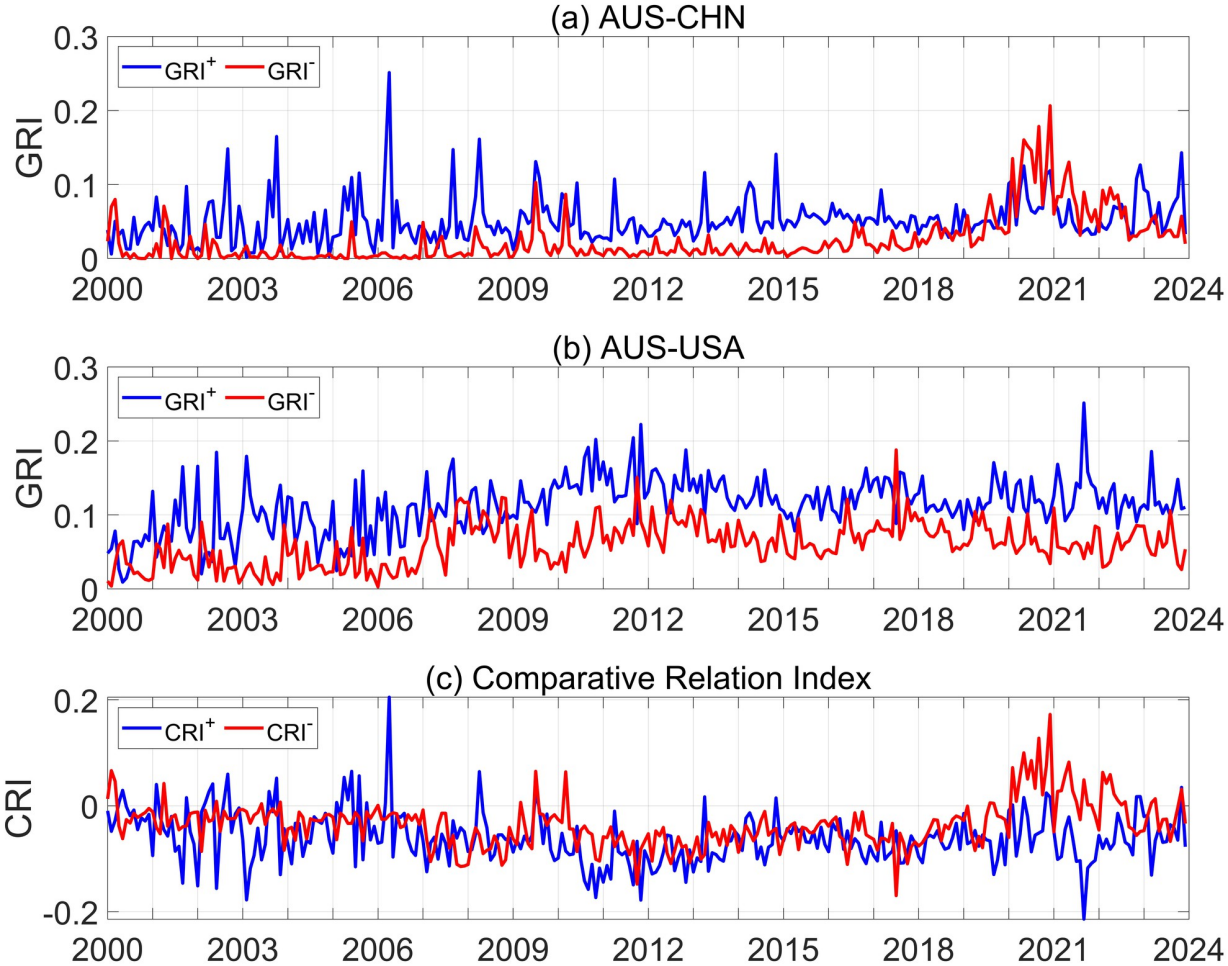
The time series of Australia’s GRI and CRI with China/the US from 2000 to 2023.

Based on the GRI, this study calculates the geopolitical relations of 19 Asia-Pacific countries with China and the United States regarding cooperation and conflicts. Australia serves as a case study to examine the evolving dynamics of its interactions with China and the US (see Fig 2), where the blue and red curves in sub-figures (a) and (b) represent the time series of GRI indicators for Australia-China and Australia–United States in cooperation and conflict, respectively, on a monthly time scale. It can be observed that the curve is filled with various peaks, which can be accurately correlated with specific events occurring between Australia and China, as well as between Australia and the United States. Consequently, the GRI constructed in this study can accurately capture the key events in the interaction between the two countries, and these results are also valid for other countries.

As depicted in subplot (a) of Fig 2, the relationship between Australia and China during Rudd’s and Gillard’s tenures was characterized by a dominant emphasis on cooperation. This is because the Australian strategy adopted can be described as a “balancing act.” The most evident manifestations of this strategy were frequent state visits and close economic cooperation between the two countries (Table 2).

**Table 2:** Summary of events and their occurrence time for Australia and China under different fields.

Type	Fields	Occurrence time	Descriptions
Cooperation	Politics	Oct 2003, Aug 2005, Apr 2006, Sep 2007, Apr 2008, Jul and Aug 2009, Oct 2009, Apr 2013, Nov 2014, Nov and Dec 2022, Sep 2023, Nov 2023	State visit or Leaders’ Meeting
	Economy	Mar 2006, Nov 2022 Apr 2023	Economic cooperation
Conflict	Politics	Jul 2009, Jul and Aug 2020, Nov 2020, Apr and Sep 2021, Mar and Jun 2022, Mar 2023	Government intervention
	Economy	May 2020, Sep 2020, Nov and Dec 2020, Apr, May and Jun 2021, Apr 2022,	Economic conflict
	Others	Apr 2010, Sep 2020	Other conflict

However, Australia’s strategy toward China significantly shifted during Morrison’s tenure (2018-2022),reflected in dramatic changes in international relationships. Specifically, the conflict-related GRI curve (red line) between Australia and China showed a significant increase from 2020, exceeding the cooperation-related curve and reaching a peak at the end of that year. This was because of a series of disputes between China and Australia. A climate of conflict gripped the relationship between China and Australia until the new Australian prime minister came to power in May 2022. Recently, the relationship between Australia and China has witnessed a gradual thaw, with the blue curve representing cooperation between the two countries consistently remaining above the red curve. Table 2 lists the types of events and their temporal distributions, which serve as tangible examples of hedging tactics.

Let us turn to the dynamic evolution of the GRI time series between Australia and the US in Subgraph (b). This indicates greater cooperation between Australia and the United States than conflict. This is because there was no genuine dispute between the two countries. Cooperation is a recurring theme in Australian-US interactions and is the result of Australia’s hedging strategy against America. In particular, the events of QUAD in March 2021 and AUKUS (a trilateral security partnership between Australia, the UK and the US) in September 2021, during Morrison’s leadership, opened fresh avenues for enhancing Australia-US collaboration. The US State Department announced that it had approved the sale of 220 BGM-109 Cruise missiles (Tomahawk) in Australia on March 16, 2023.

We then discuss Australia’s interactions with China and the US based on the CRI, Fig 2(c). The blue curve in sub-graph (c) is obtained by subtracting the blue curve in sub-graphs (a) and (b), whereas the red curve in sub-graph (c) is derived from the red one. Since 2020, the *CRI*^−^ has been mostly positive whereas the *CRI*^+^ has been predominantly negative, which, by the definition of the CRI, indicates that Australia is significantly closer to the US over this period. This coincided with a fundamental shift in strategy toward China and the US during Morrison’s tenure. However, as of 2020, most of Australia’s CRI with China and the US fell into quadrants I and III, largely because Australia implemented a hedging strategy aimed at balance. The effects of Australia’s hedging strategy with Sino-US have significant temporal heterogeneity. Therefore, we argue that a combination of the four-quadrant model and GDELT data provides a quantitative assessment of the effects of Australia’s China-US hedging strategy.

### 4.2 The spatio-temporal heterogeneity of hedging strategies’ effects for Asia-Pacific states with Sino-US

#### 4.2.1 Categorization of Asia-Pacific countries based on the implementation effect of hedging strategies

Fig 2(c) demonstrates significant differences in hedging strategies across different periods. Similarly, each state in the Asia-Pacific region follows its hedging strategy logic, and the results for individual states are highly different. In other words, there are huge differences in the hedging strategies of Asia-Pacific countries against China and the US, the actual effects of these strategies will naturally have temporal-spatial heterogeneity. To quantify the extent of the effects of hedging strategies, we perform a two-step transformation of the CRI indicator according to the four-quadrant framework.

**Fig 3:**
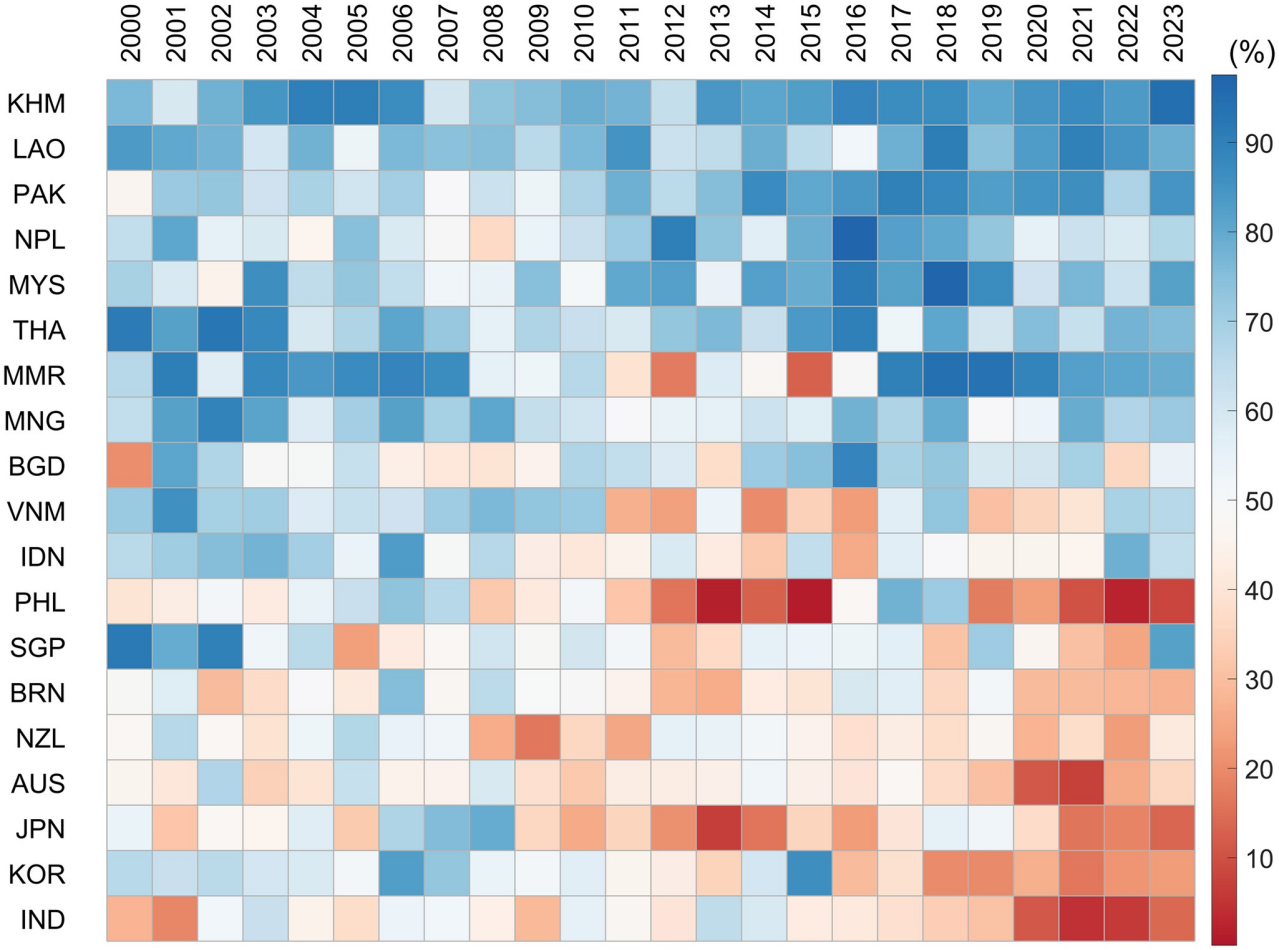
Spatio-temporal atlas of the relational proximity between Asia-Pacific countries and China.

The first step is to convert the CRI of cooperation and conflict into a percentage of closeness with China using the algorithm mentioned in the theoretical framework. In the second step, because the monthly frequency is unsuitable for illustrating the temporal-spatial heterogeneity of hedging strategies’ effects, the arithmetic mean is used to transform the monthly results into annual ones. We then plotted a color block diagram of the degree of the hedging tactics’ effects for Asia-Pacific countries with China and the US from 2000 to 2023, as depicted in Fig 3. Higher values indicated a relatively close relationship with China. In contrast, this implies a relatively closer relationship with the US.

Careful observation of the dynamic evolution of the relative relations between these Asia-Pacific countries and China shows that the temporal-spatial heterogeneity will become more pronounced as tensions between China and the US escalate, and the pace of change in the effects of some countries’ strategies will also accelerate significantly. Interestingly, the 19 Asia-Pacific countries can be roughly divided into three categories according to the dynamic evolution characteristics of the hedging strategy effects. In conjunction with the hedging strategies adopted by these countries, the following three categories are defined: significantly closer to China, significantly closer to the US, and Swinging. Considerable variations exist in the effects of hedging strategies across countries, even within the same category. Thus, a comprehensive examination is conducted for each category.

*1. Group1—Significantly closer to China*. Our four-quadrant framework indicates that Cambodia (KHM), Laos (LAO), Pakistan (PAK), Nepal (NPL), Malaysia (MYS), Thailand (THA), Myanmar (MMR), and Mongolia (MNG) are significantly closer to China between 2000 and 2023. Interestingly, the degree of effects of hedging strategies employed by these countries with China and the US varies. To illustrate this, we consider Cambodia and Malaysia. Malaysia and Cambodia are both qualified as light hedgers, given their level of deference to China and the diversification of their foreign relations [68]. First, Cambodia has no border or territorial disputes with China and wants to develop its economy without imposing political conditions on investment projects. Consequently, Cambodia seeks to prioritize its economic gain by engaging with China [68]. Cambodia maintained a certain degree of political and economic cooperation with the US. However, the effects of Cambodia’s hedging strategy are significantly closer to those of China, as shown in Fig 3. From 2000 to 2023, the average value of the effects of Cambodia’s hedging strategy exceeded 80%. Second, in strategic competition between the United States and China, Malaysia, a middle-power country, has adopted a light-hedging strategy to mitigate risk and maximize opportunities. This strategy involves direct engagement, limited balancing, and band wagoning. The features of this strategy are an insistence on not taking sides, the concurrent adoption of open deference and indirect defiance, and an active effort to cultivate a fallback position [69]. With the uncertainty of the US commitment and economic reliance on China, Malaysia narrowed its distance from China after implementing a hedging strategy, with an average level of approximately 70%.

*2. Group2—Significantly closer to the US*. Philippines (PHL), Australia (AUS), Japan (JPN), South Korea (KOR), India (IND), New Zealand (NZL), and Brunei (BRN) can be considered significantly closer to the US. Australia, and New Zealand, which belong to the Five Eyes Alliance. In contrast, Japan and South Korea have a mutual defense treaty with the US. The average effects of these states’ hedging strategies with China from 2000 to 2023 are approximately 40%, 43%, 38% and 47%, respectively. Although not a conventional ally, India’s multilateral relations with China and Pakistan, its position and potential in South Asia, and its vigilance against China’s rise have gradually made India a key target for US efforts to contain China. The historic transformation of US-India relations began during Clinton’s tenure (1993-2001), made substantial breakthrough under the Bush administration (2001-2009), and continued to consolidate and strengthen during Obama’s administration (2009-2017). Based on continuing the policy of previous US governments toward India, the Trump administration attaches greater importance to India’s strategic position. From 2000 to 2023, the average value of the effects of India’s hedging strategy on China was approximately 37%.

Regarding the Philippines, then-president Gloria Macapagal Arroyo (2001–2010) started engaging China to reduce the Philippines’ dependence on the United States, but without fundamentally undermining the Philippines–US strategic alliance [43]. Fig 3 shows a significant improvement in the effects of hedging strategies between the Philippines and China during this period. However, the new administration (2010–2016) under Aquino decided to stay away from close engagement with China after Arroyo stepped down amid corruption scandals [43]. Meanwhile, it deepened its alliance with the US and defined its goal as countering China’s potential threat. This transition is illustrated in Fig 3. Since Rodrigo Duterte took office in 2016, the new administration announced a “strategic separation” from its treaty ally (the US). It adopted a restrained approach to the Hague Tribunal’s arbitration ruling [43]. This finding is consistent with the results of our quantitative analysis.

*3. Group3—Swinging*. Singapore (SGP), Indonesia (IDN), VietNam (VNM) and Bangladesh (BGD) can be classified as swinging type. For Singapore, it has been sending both supportive and critical signals to both great powers and never allowing itself to be too closely associated with either [34]. During the last three decades, Singapore has repeatedly stressed its commitment to neutrality between Washington and Beijing [70]. For Indonesia, Maximizing benefits and mitigating risks have become major themes in Indonesia’s foreign policy. An Indonesian-conceived plan called the ASEAN Outlook on the Indo-Pacific (the Outlook) was adopted by the Association of Southeast Asian Nations (ASEAN) on 23 June 2019. Jakarta emphasizes inclusivity in the Outlook document, meaning it is taking a middle path, calling for a free, open and inclusive Indo-Pacific region [71]. In September 2021, Indonesian Foreign Minister Retno Lestari Priansari Marsudi publicly stated that Indonesia did not want to take sides in the Sino-US conflict and was ready to cooperate with the US. The return of great power rivalry and the resulting uncertainty that it has engendered in the Indo-Pacific region has reinforced the importance of Indonesia’s free and active foreign policy doctrine and non-alignment principle, ensuring that Jakarta is not forced to choose between supporting China or the US [72]. From a theoretical perspective, Indonesia’s policies in the face of China’s rise, intensifying US-China rivalry, and regional uncertainty tend to be eclectic [72], which is consistent with our quantitative results. Vietnam voiced its concerns about China over the US while trying to maintain close political and economic ties with the country. Officials in Hanoi said China’s action is a “destabilizing factor and a direct challenge to the US presence in the region”. The year 2017 witnessed new enhancements in US–Vietnam strategic cooperation [43]. According to the calculations, the average value of the effects of the three countries’ hedging strategies against China and the US from 2000 to 2023 is approximately 50%. Notably, the China-Vietnam territorial dispute in 2014 threw bilateral relations to their lowest point since 1991, which is clearly reflected in Fig 3.

#### 4.2.2 Dynamic evolution of the implementation effect of hedging strategies in different periods

**Fig 4:**
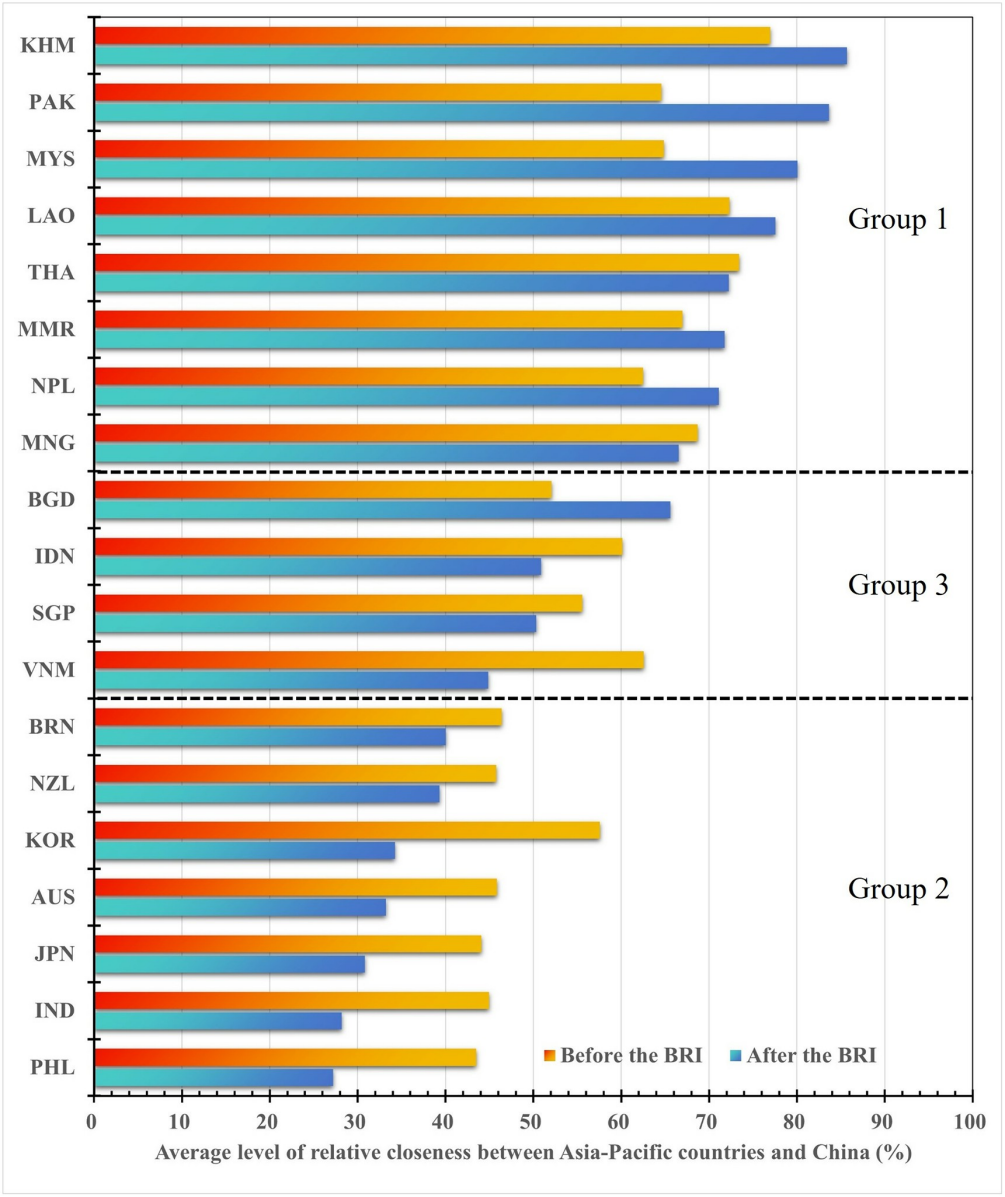
The comparison of the average level of relative closeness between Asia-Pacific countries and China before and after the implementation of the Belt and Road Initiative.

The diplomatic strategies of China and the US greatly influence the hedging strategies of countries in the Asia-Pacific region. Here we initially investigates the effects of Asia-Pacific countries’ hedging strategies toward China and the US before and after implementing the Belt and Road Initiative (BRI). In response to the BRI, the US government has adopted a series of strategies in the Asia-Pacific region, including Indo-Pacific strategy, QUAD, and IPEF. Asia-Pacific countries need to adjust their strategies to cope with changes in Sino-US relations. Based on the results in Fig 3, we calculated the average annual relative closeness between Asia-Pacific countries and China from 2000 to 2013 and from 2014 to 2023, as shown in Fig 4. Since implementing the BRI, the average relative degree of countries closer to China has improved significantly, particularly in Cambodia, Laos, Pakistan, Nepal, Malaysia, and Myanmar. Meanwhile, the average degree of relative closeness between China and the group of countries closer to the US shows a significant decline, implying that these countries were moving relatively far away from China. In addition, there have been subtle changes in swinging countries. Indonesia and Vietnam have significantly lower average degrees of relative closeness to China, reflecting that these two countries are moving away from China compared to before 2013, while the opposite is true for Bangladesh, which is moving closer to China.

**Fig 5:**
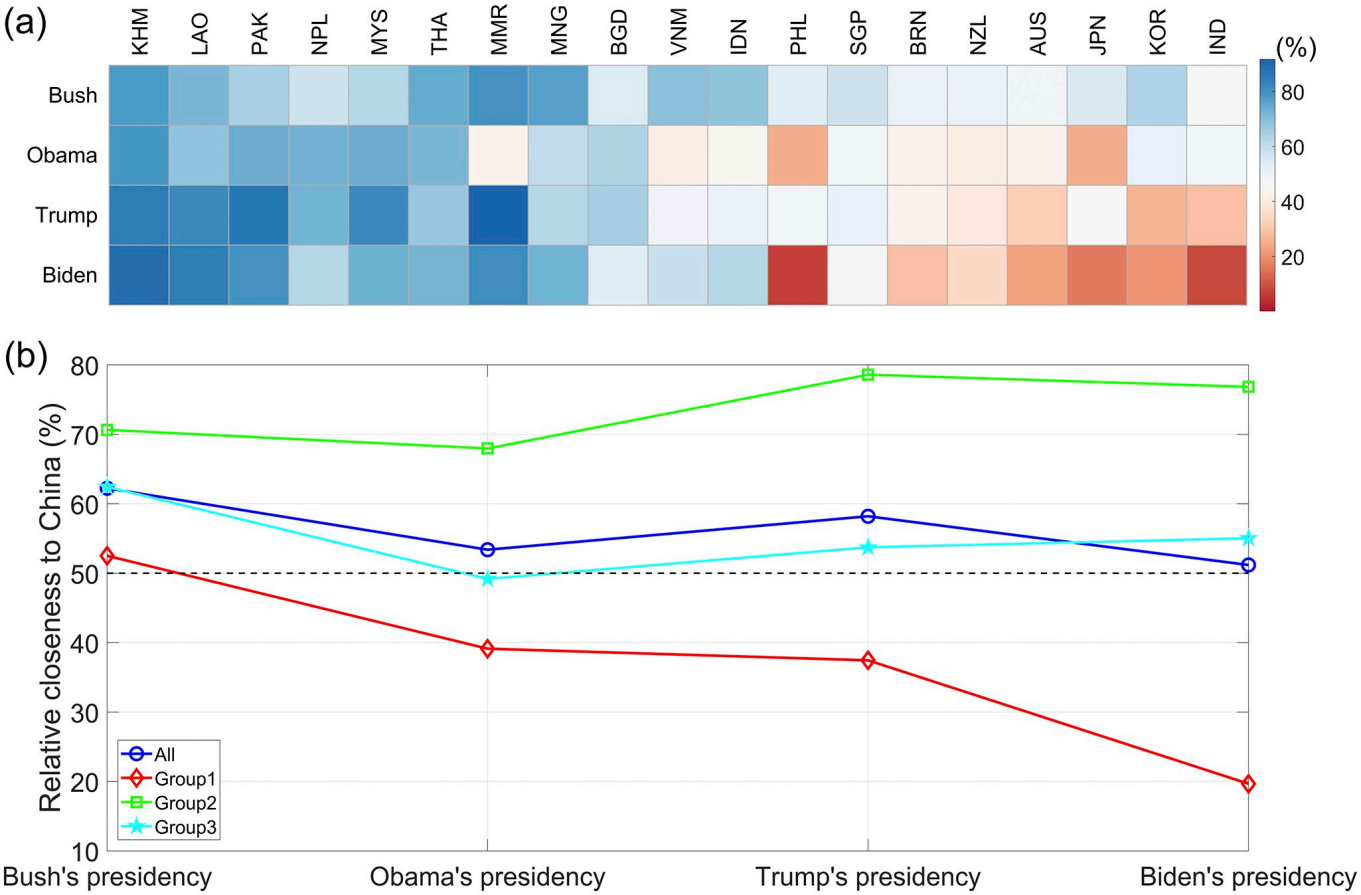
The comparison of the average degree of relative closeness between Asia-Pacific countries and China under different US presidencies.

We then examine the varying degrees of the relative relationships that Asia-Pacific countries maintain with both China and the US across different presidential administrations in the US. Based on the two-step transformation, we calculate the relative relationship distance of the Asia-Pacific countries with China during the presidencies of George W. Bush, Barack Obama, Donald Trump, and Joe Biden, as illustrated in Fig 5. During President Bush’s administration, the US primarily focused on counter-terrorism efforts in the Middle East, resulting in a lack of pronounced strategic competition with China and fostering an environment of strategic cooperation. The average relative closeness between Asia-Pacific countries and China during this period was 62.23%, with the average distance for seven countries that are significantly closer to the US being 52.51%. Under President Obama, the US adopted a strategy aimed at re-engagement with the Asia-Pacific region, marking a significant shift in strategic focus towards Asia. This pivot had a considerable impact on the hedging strategies of Asia-Pacific nations, evidenced by a notable decrease in their relative closeness from China, particularly among countries closely aligned with the US as shown in Fig 5(b).

During President Trump’s administration, the US withdrew from Trans-Pacific Partnership Agreement (TPP) and implemented a free and open Indo-Pacific strategy. Concurrently, China’s BRI advanced rapidly, intensifying strategic competition between the two powers. These fluctuations resulted in an increase in the average relative closeness between Asia-Pacific countries and China, as shown in Fig 5(b). Notably, the relative relation distance between countries closely aligned with the US decreased, while the distance for countries more aligned with China increased significantly. The relative closeness for swing countries remained stable at approximately 50%. In the current Biden administration, the US has continued the Asia-Pacific strategy initiated by Trump and has introduced the QUAD and AUKUS. Both AUKUS and its closely related counterpart, the Quad, have been viewed as a means of strengthening US regional ties. AUKUS is not conducive to the preservation of peace in Asia and beyond [73]. The strategies employed by the US to revitalize multilateral partnership structure and restore relationships with allies in the Asia-Pacific region, have led to a significant reduction in the average relative closeness between countries that are significantly closer to the US and China, while there has been little change in the relationships of countries that are significantly closer to China and those classified as swing countries.

By comparing the effects of hedging strategies adopted by Asia-Pacific countries during four American presidential terms, it becomes clear that the relative proximity of these countries to China has generally decreased. Specifically, those countries that align more closely with the US have experienced a consistent and significant decline in their relationship with China. In contrast, nations that maintain closer ties with China have observed an upward trend in their interactions. Furthermore, countries that fluctuate between these two influences have displayed a “V”-shaped pattern, with a notable increase in their closeness to China since the Obama presidency.

Fundamentally, the strategic balance in Asia will be defined by how the pervasive tensions and competition between China and the US play out [74]. Besides, major international events will also have an impact on the strategic choices of countries in the Asia-Pacific region in relation to China and the United States. Taking the Russia-Ukraine conflict as an example, although geographically limited to Eastern Europe and the Black Sea, the war in Ukraine is a major inter-state conflict that is likely to have long-term global ramifications, including for the Asia-Pacific [75]. Asia-Pacific countries generally maintained a neutral position on the crisis, and many countries expressed support for a peaceful resolution of the conflict through diplomatic means [76]. The US has leveraged this crisis to integrate its allies in the Asia-Pacific region, promote strategic cooperation and coordination, and intensify the strategic competition between China and the US. This may result in increased pressure on the hedging strategies of countries in the Asia-Pacific region about their relationships with China and the US. This will have significant implications for the strategic approaches of Asia-Pacific countries in navigating the strategic competition between China and the US. Several Asia-Pacific countries have made significant alterations to their strategic decision-making processes, and a series of unparalleled indications have emerged. To illustrate, following the eruption of the Russia-Ukraine conflict, Japan is striving to diversify its regional alliances within the context of its alliance with the US [77]. Discussions of “nuclear sharing”—allowing nonnuclear weapons states to partake in planning around nuclear weapons and operating nuclear missions—were raised in Japan for a time [78].

Furthermore, concerning the stability of the regional structure, regionalism provides a voice to weaker actors, with ASEAN serving as a prime example. ASEAN plays a pivotal role in ensuring regional stability within Southeast Asia and the wider Asia-Pacific region [44]. The organization aspires to uphold its centrality, aiming to dominate the discourse surrounding multilateral institutions and regional cooperation in the Asia-Pacific or Indo-Pacific region, while engaging with all external powers without exhibiting bias. However, the intensification of great power competition increasingly challenges this centrality [44]. Our results indicate that certain ASEAN member states have moved more closely with China, while others have gravitated towards the US; even those nations attempting to maintain a neutral stance find it difficult to remain uninvolved. Should great powers overlook the importance of regionalism and disregard the perspectives of weaker states in the Asia-Pacific—by heightening competition and pressuring ASEAN nations to take sides—this will jeopardize ASEAN’s central role, ultimately threatening both regional stability and the global order. In response to these challenges, some Chinese scholars have put forward suggestions, such as Zhao’s *Tianxia* concept, which includes inclusiveness and harmony, promoting the ontology of co-existence and relational rationality hand in hand with rational risk aversion in a globalized world [79].

## 5 Discussion

As the strategic rivalry between China and the US intensifies, Asia-Pacific countries face significant challenges in making strategic adjustments and diplomatic choices under the influence of these two major powers. These nations’ strategic decisions concern their interests and potentially affect major country relations and global/regional stability. Therefore, nations in the Asia-Pacific region have adopted hedging strategies to reduce or avoid the consequences of Sino-US strategic competition and to maximize opportunities. However, evaluating the effects of the strategies adopted by states in international affairs is a long-term challenge in international politics. Fortunately, the massive GDELT event database is a valuable resource for empirical studies, facilitating the quantification of existing political phenomena. This study proposes a novel four-quadrant evaluation framework based on geopolitical relations, which expands the international relations theory. By modifying the key indicator in the GDELT datasets, we constructed the Geopolitical Relation Index, which can quantitatively describe the geopolitical relations between any two countries in terms of both cooperation and conflict and the Comparative Relation Index, which can be used to evaluate the effects of hedging strategies of third-party with two great powers. The effects of hedging strategies in 19 Asia-Pacific countries against China and the US since 2000 were dynamically quantified by integrating theoretical models with these two indices. We uncovered three interesting findings after considering the dynamic changes in these Asia-Pacific nations in different periods.

First, the effects of the hedging strategies adopted by Asia-Pacific countries under strategic competition between China and the US exhibit significant spatiotemporal heterogeneity. This heterogeneity becomes more prominent during tense periods in Sino-US relations, with the speed of change in the effects of some countries’ strategies, such as Singapore, Vietnam, and Indonesia. These dynamic strategies are guided by the key principles of maximizing benefits and mitigating risks. Additionally, significant differences in the actual effects of hedging across 19 countries in the Asia-Pacific region were observed over the same period. For example, in 2010, countries such as Cambodia, Laos, Pakistan, Nepal, Malaysia, Thailand, and Myanmar had closer ties with China, while Indonesia, the Philippines, Singapore, Australia, Japan, and South Korea leaned toward the US.

Second, based on the dynamic evolutionary characteristics of the hedging strategies, the 19 Asia-Pacific countries can be roughly categorized into three groups: significantly closer to China, significantly closer to the US, and swinging. Countries such as Cambodia, Laos, Pakistan, Nepal, Malaysia, Thailand, Myanmar, and Mongolia lean toward China, whereas the Philippines, India, Japan, Australia, South Korea, New Zealand, and Brunei lean toward the US. Notably, India, Australia, and New Zealand are US allies, reflecting their preferences for political and military cooperation with the US. Singapore, Indonesia, Vietnam, and Bangladesh exhibit a “swinging” type, with the average degree of hedging effects was approximately 50% from 2000 to 2023. This finding is inseparable from the fundamental principles of hedging strategies.

Third, by analyzing the effects of the hedging strategies implemented by Asia-Pacific nations against China and the US across the four terms of US presidents and before and after China’s BRI, some very interesting phenomena were discovered. On the one hand, since the implementation of the BRI, concerning China or the US, relatively close countries have become closer, while those farther away have drifted even further. Concretely, countries more inclined to support China have tightened their ties with China. Among them, Cambodia, Laos, Pakistan, Nepal, Malaysia, and Myanmar show a clear performance, with the closeness to China increasing significantly. Meanwhile, countries relatively leaning to the US have been forging closer relationships with the US. The Philippines, India, Japan, Australia, South Korea, New Zealand, and Brunei are far away from China. Moreover, swing countries have also undergone subtle changes, with Indonesia and Vietnam moving further away from China than before 2013, while the opposite is true for Bangladesh, which is moving closer to China. On the other hand, those countries that align more closely with the United States have experienced a consistent and significant decline in their relationship with China. In contrast, nations that maintain closer ties with China have observed an upward trend in their interactions. Furthermore, countries that fluctuate between these two influences have displayed a “V”-shaped pattern, with a notable increase in their closeness to China since the Obama presidency. These changes are largely closely related to a series of diplomatic strategies adopted by the US or China.

These findings demonstrate that our theoretical framework and quantitative methods not only aid in evaluating and monitoring the execution effects of Asia-Pacific countries’ diplomatic strategies toward China and the US but also provide guidance for timely adjustments in foreign policies, contributing positively to sustainable development in the Asia-Pacific region. The academic contributions of this study are as follows. First, the innovative proposal of a four-quadrant analysis framework and corresponding quantitative methods advance the field of geopolitical sciences. Second, this study explores a new approach to combining theoretical models with big data, facilitating a dynamic analysis of international relations, and overcoming traditional research methods’ subjectivity, one-sidedness, and static nature. Third, to the best of our knowledge, this is the first study in which a dynamic and quantitative assessment of the effects of hedging strategies toward China and the US in Asia-Pacific countries has been provided. The results also carry implications for practical applications. For countries in the Asia-Pacific region, the results facilitate a dynamic evaluation of the effect of hedging strategies and offer guidance for necessary strategic adjustments. For both China and the US, the research enables a timely understanding of the responses from Asia-Pacific nations.

This article constitutes a novel endeavor to quantitatively evaluate the effects of implementing hedging strategies; nonetheless, there are several areas that warrant further enhancement. In subsequent research, we intend to improve our study in three principal dimensions. First, we will strive to create sub-indices that incorporate political, economic, security, and military factors to facilitate a more thorough assessment of the implications of hedging strategy implementation. Second, we will analyze in detail the adjustments in the hedging strategies of Asia-Pacific countries, focus on the motivations behind them, and put forward constructive suggestions. Third, we will examine the impact of the implementation of hedging strategies among China, the US and Asia-Pacific countries on international trade and investment, and quantify the hedging effect networks within the region.
